# Improved Mechanical and Tribological Properties of Metal-Matrix Composites Dispersion-Strengthened by Nanoparticles  

**DOI:** 10.3390/ma3010097

**Published:** 2009-12-29

**Authors:** Evgenii Levashov, Victoria Kurbatkina, Zaytsev Alexandr

**Affiliations:** Moscow Institute of Steel and Alloys, Leninskii pr. 4, Moscow, 119049 Russia; E-Mails: levashov@shs.misis.ru (E.L.); vvkurb@mail.ru (V.K.)

**Keywords:** metal-matrix composites, nanoparticles, dispersion strengthening, diamond tools

## Abstract

Co- and Fe-based alloys produced by powder technology are being widely used as a matrix for diamond-containing composites in cutting, drilling, grinding pplications, etc. The severe service conditions demand that the mechanical and tribological properties of these alloys be improved. Development of metal-matrix composites (MMCs) and alloys reinforced with nanoparticles is a promising way to resolve this problem. In this work, we have investigated the effect of nano-sized WC, ZrO_2_, Al_2_O_3_, and Si_3_N_4_ additives on the properties of sintered dispersion-strengthened Co- and Fe-based MMCs. The results show an increase in the hardness (up to 10 HRB), bending strength (up to 50%), wear resistance (by a factor of 2–10) and a decrease in the friction coefficient (up to 4-fold) of the dispersion-strengthened materials. The use of designed alloys as a binder of cutting diamond tools gave a 4-fold increment in the service life, without reduction in their cutting speed.

## 1. Introduction

Co- and Fe-based alloys produced by powder technology are seeing wide use as a matrix for diamond-containing composites employed in cutting, drilling, grinding applications, *etc.* [1,2,3]. The cutting ability of diamond segments is known [4,5,6,7,8,9,10,11] to depend markedly on the mechanical, physicochemical, and tribological properties of the matrix material (binder). Severe service conditions (intense hydroabrasive wear, impact stresses, and elevated temperature in the cutting area) demand that the mechanical and tribological properties of binders be improved. Development of new metal matrix composites and alloys reinforced with nanoparticles is a promising way to resolve the problem [12,13,14,15,16,17,18,19,20,21,22,23,24]. The use of nano-sized particles (instead of micro-sized ones) for reinforcement of hard compounds is advantageous for the following reasons. (1) according to the Orovan equation, the effectiveness of dispersion strengthening depends [15] on the particle size of embedded particulates, so that a relatively low amount of reinforcing phase (below 5 vol %) can be expected to markedly improve the mechanical properties of reinforced alloys. (2) The chemical activity of nanoparticles is known to be higher than that of bulk material due to better interparticle contact between the components. Interaction between the nanoparticles and diamond grains also improves the adhesion of binder to diamond and hence the tool life.

In the first section of this paper, we will describe the preparation of composite materials with uniform distribution of reinforcing particles and the effect of embedded nanoparticles on the sintering process and on mechanical/tribological properties of alloys. In the second section, we will report on the applications of the designed alloys as a binder for diamond tools.

## 2. Results and Discussion

### 2.1. Optimization the intermixing process

**Figure 1:**
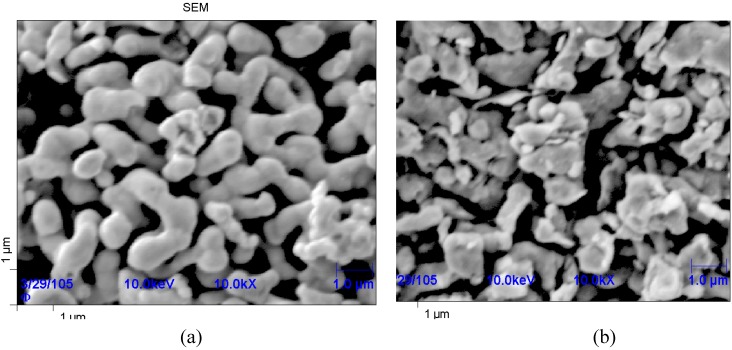
Morphology of (a) initial Co powder and (b) Co + WC mixture after intermixing.

Uniform distribution of nanoparticles in a charge is a key factor that defines the effectiveness of dispersion hardening. In case of nanoparticles it is very difficult to attain because the duration of intermixing is known to depend exponentially on the size of the mixed particles. In this work, intermixing was carried out in a centrifugal planetary mill (CPM). Figure 1 shows the shape of Co particles before and after treatment in the mill. After intermixing, the Co particles are seen to acquire a disk-like shape.

**Figure 2:**
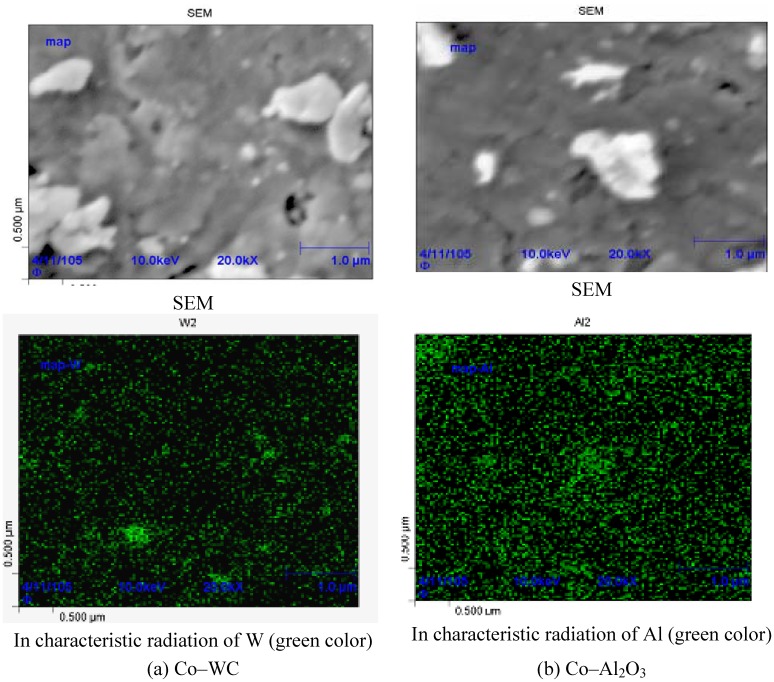
SEM images and Auger maps of (a) Co–WC and (b) Co–Al_2_O_3_ mixtures.

Figure 2 shows the SEM images and Auger maps of intermixed charges containing nanoparticles.

**Figure 3:**
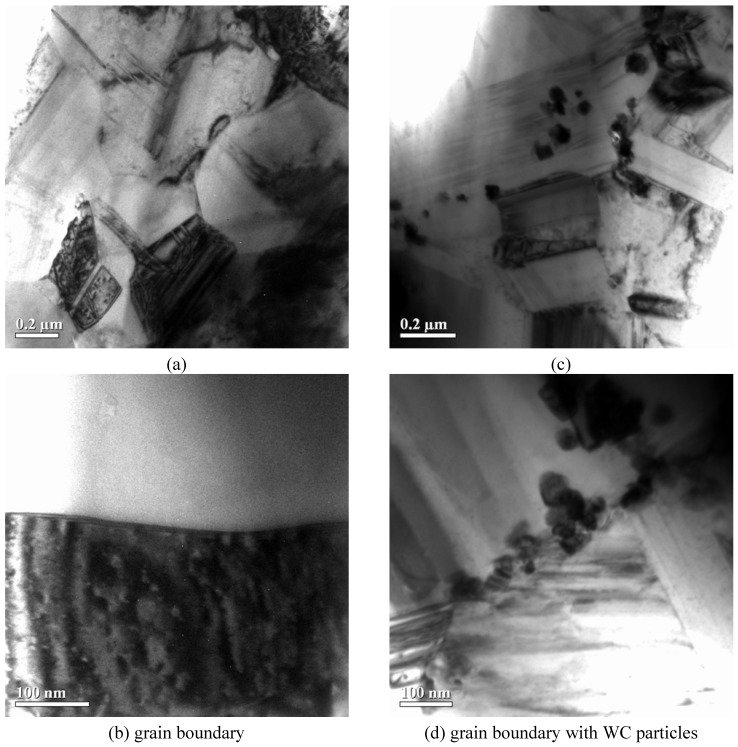
TEM images of sintered (a, b) pure Co and (c, d) Co–WC alloy.

**Figure 4:**
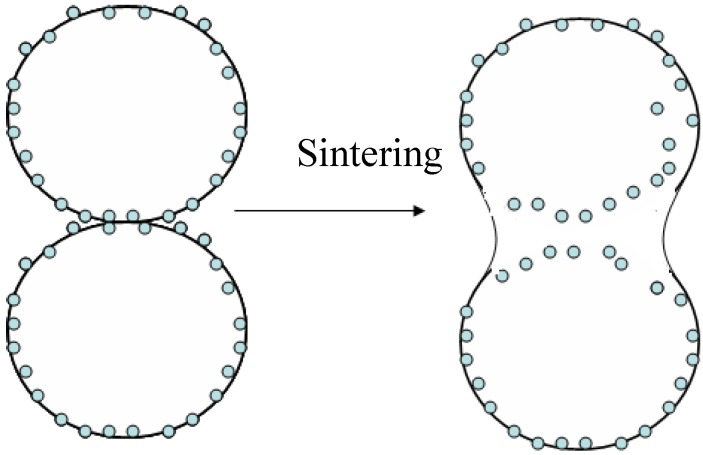
Schematic of nanoparticles insertion into the grain body during sintering.

As follows from the Auger maps in Figure 2, the chosen intermixing mode provided a rather uniform distribution of nanoparticles over the charge. It is desirable that such a distribution in starting powder mixtures be inherited by a sintered product. For TEM investigation of sintered samples, we prepared Co–WC foils (Figure 3). The grain size in sintered pure Co is 0.2–1.5 μm (Figure 3a,b). Figure 3c,d show the size of WC particles and their distribution in the sample produced under the same conditions as pure Co. It is important to note that nanoparticles have been found not only at the grain boundary, but also in the grain body (Figure 3c). This can happen as a result of the following two processes: (1) the nanoparticles initially situated on the grain boundary are encapsulated onto the grain body, as shown in Figure 4. (2) Nanoparticles are hammered into the Co grains during intermixing as a result of frequentative collisions of grinding bodies (steel balls) with powder particles.

### 2.2. Mechanical and tribological properties of hot-pressed samples dispersion-strengthened with nanoparticles

Table 1 shows the mechanical properties of the sintered samples dispersion-strengthened (DS) with nanoparticles prepared in a DSP-1 hot-pressing installation (860 °C, 350 kg/cm^2^, inert atmosphere).

**Table 1:** Mechanical properties of hot-pressed DS samples.

Composition, wt %	*T*_s_, °C	ρ, g/cm^3^	Porosity, %	Hardness, HRB	σ_b_, MPa	KCU, J/cm^2^
Co	880	8.64	2.9	105	1150	4.6
Co "0", τ_mix_ = 3 min	880	8.40	5.7	107	1560	3.3
Co–6% WC	900	8.52	6.0	105	1140	3.4
Co–2% WC	900	8.49	6.0	106	1360	4.2
Co–6% W	900	8.04	13.3	97	790	3.2
Co–0.92% Al_2_O_3_	900	8.29	5.8	107	988	3.69
Co–3.3% Al_2_O_3_	900	7.12	16.1	97	140	2.3
Co–1.13% ZrO_2_	900	8.44	1.9	106	970	4.3
Co–2.56% ZrO_2_	900	8.34	5.4	110	1230	3.2
Co–4.53% ZrO_2_	900	7.69	11.3	104	1060	2.5
V21	845	7.89	3.0	89	890	3.76
V21 "0"	845	7.78	4.2	91	960	3.48
V21–1% Al_2_O_3_	860	7.74	3.8	102	910	2.72
V21–2% Al_2_O_3_	860	7.65	4.0	103	690	2.46
V21–3.3% Al_2_O_3_	860	7.74	4.2	104	900	2.09
V21–1.3% ZrO_2_	860	7.84	3.1	99	990	3.01
V21–2.9% ZrO_2_	860	7.77	3.2	106	770	2.57
V21–5% ZrO_2_	860	7.68	3.5	104	660	2.63
V21–2% WC	860	7.94	3.3	104	1070	4.04
V21–4% WC	860	7.98	3.6	103	1050	4.11
V21–6% WC	860	8.04	4.0	102	1370	3.03
B13	865	8.30	4.0	97	870	4.2
B13 "0"	865	8.21	5.1	107	1060	3.8
B13–6% WC	880	8.24	7.3	100	900	3.0
B13–4% WC	880	8.22	6.7	105	1170	3.9
B13–2% WC	880	8.29	5.0	105	1020	3.5
B13–2.6% ZrO_2_	880	8.11	4.9	101	740	2.8
B13–1.3% ZrO_2_	880	8.14	5.2	106	750	3.4
B13–1.3% (ZrO_2_–5% Y_2_O_3_)	880	8.16	5.0	105	850	3.7
B13–2% Al_2_O_3_	880	7.84	7.2	99	570	2.7
B13–1.6% Si_3_N_4_	880	8.00	4.9	105	600	3.8
B13–0.8% Si_3_N_4_	880	8.12	4.8	106	810	3.5

Here Co stands for Co-based binder (Co extra fine), V21 for Fe-based binder Diabase V21 (Fritzsch), and B13 for Cu–Ni binder B13. V21 "0", Co "0", B13 "0" pure binders after PTM treatment.

**Figure 5:**
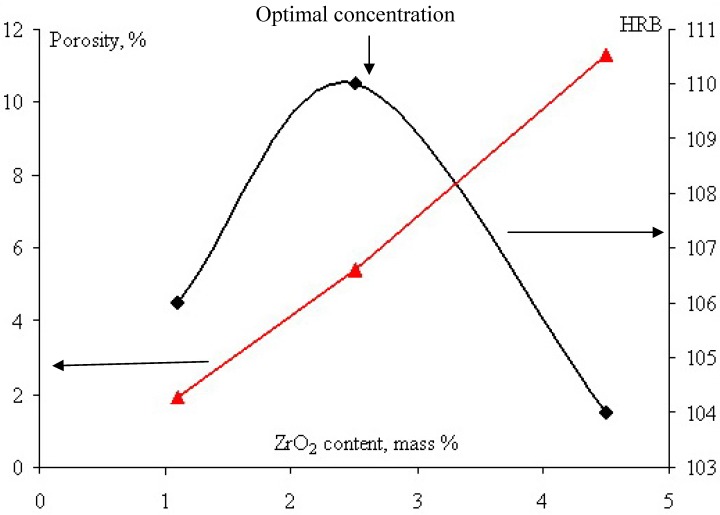
Porosity and HRB hardness as a function of the ZrO_2_^nano^ content of sintered samples.

As follows from Table 1, the residual porosity of sintered pure samples does not exceed 4%, while that of DS samples may attain values of up to 5–16%. With an increasing amount of added nanoparticles, the porosity grows. It turns out that the properties of sintered samples are affected by two opposing factors: The reinforcement with embedded nanoparticles and a weakening caused by increasing porosity. As a result, there is an optimal concentration of nanoparticles, as shown in Figure 5.

The bending strength (σ_b_) and wear resistance (*W*) as a function of the amount of embedded nanoparticles also exhibited a maximum. These observations are in agreement with theoretical predictions. Impact straight reduction explains the fact that the pores (especially sharp-edged) act as stress concentrators facilitating crack propagation.

Some results of tribological testing are given in Table 2. The friction coefficient of DS alloys is close to that of pure alloys. The wear resistance shows good correlation with the mechanical properties (Table 1). The wear resistance of the alloys with optimal concentration of nanoparticles increases by a factor of up to four in case of Co and V21 binders. The effect is still more pronounced in case of the B13 binder.

**Table 2:** Wear resistance (*W*) and friction coefficient (μ) of DS alloys.

Composition, wt %	μ	*W*, mm^3^/(N m) × 10^–5^
Co	0.68	1.72
Co "0"	0.84	1.30
Co–2% WC	0.89	0.61
Co–6% WC	0.68	0.28
Co–6% W	0.77	0.41
Co-0.92% Al_2_O_3_	0.66	0.77
Co–3.3% Al_2_O_3_	0.76	17.08
Co–1.13% ZrO_2_	0.63	0.56
Co–2.56% ZrO_2_	0.82	1.56
Co–4.53% ZrO_2_	0.77	2.52
V21	0.67	1.33
V21 "0"	0.64	1.73
V21–1% Al_2_O_3_	0.69	1.47
V21–2% Al_2_O_3_	0.68	2.3
V21–3.3% Al_2_O_3_	0.66	1.00
V21–1.3% ZrO_2_	0.65	1.64
V21–2.9% ZrO_2_	0.67	0.92
V21–5% ZrO_2_	0.68	1.33
V21 + 2 % WC	0.64	1.10
V21 + 4 % WC	0.98	0.77
V21 – 6 % WC	0.79	0.34
B13	0.82	4.82
B13 "0"	0.73	0.481
B13–6% WC	0.93	0.609
B13–4% WC	0.74–0.89	0.096
B13–2% WC	0.84	0.274
B13–2.6% ZrO_2_	0.71	6.86
B13–2 % Al_2_O_3_	0.81	9.51
B13–1.6 % Si_3_N_4_	0.72–0.86	0.059

### 2.3. Effect of nanoparticles on the sintering process

**Figure 6:**
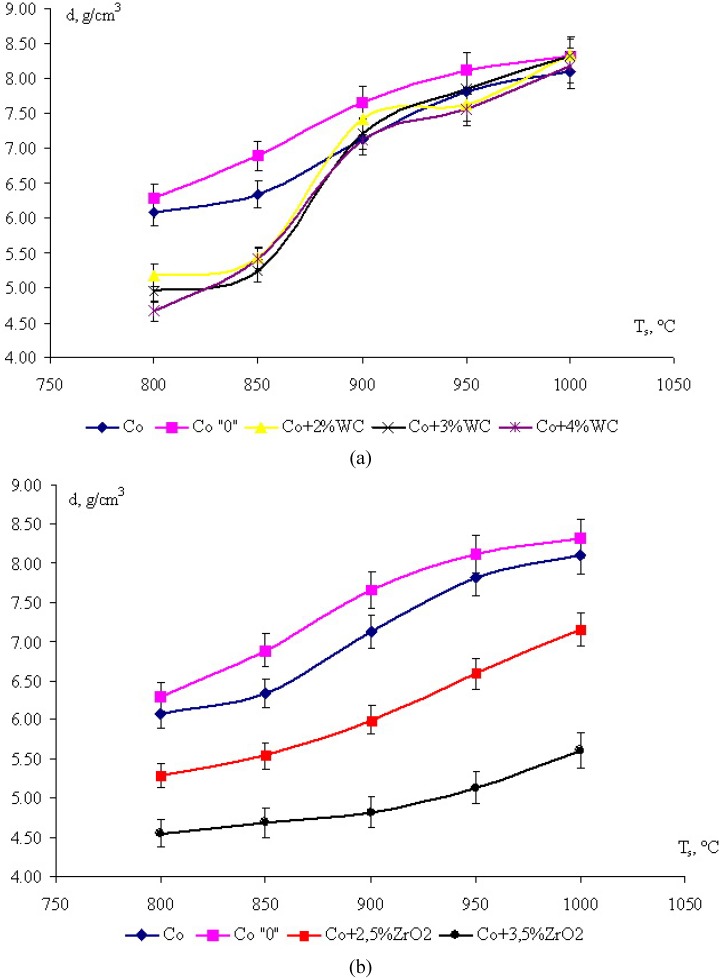
Sintering curves for (a) Co–WC^nano^ and (b) Co–ZrO_2_^nano^ mixtures (*t*_s_ = 3 min).

As mentioned in Section 2.1, CPM treatment gave mixtures with uniform nanoparticle distributions. Arrangement of nanoparticles of refractory compounds in the contact region of binder particles exerted a marked influence on the compaction kinetics during the course of solid-phase sintering. We have investigated the sintering of two systems: (1) Co–WC^nano^, in which interaction between WC^nano^ and Co is possible [25,26] and (2) Co–ZrO_2_^nano^, in which ZrO_2_ is inactive with respect to the Co matrix [25]. Figure 6 shows the sintering curves for these two systems. Porosity of the samples with ZrO_2_^nano^ is higher than of those containing WC^nano^. It our opinion, a increase in the porosity in case of inactive nanoparticles can be explained by partial blocking of the interface between matrix particles by nanoparticles, which creates an additional diffusion barrier in the course of sintering. An increase in the concentration of nanoparticles leads to their aggregation and accumulation of conglomerates in the porous interparticle space in the binder, which exerts a decelerating effect on the compaction process. Therefore, as the content of nanoparticles increases, the density of the sintered briquette decreases. CPM treatment of Co powder increases its activity in the sintering process due to mechanical activation. The density of the samples sintered in the presence of WC particles is close to that of pure Co, which explained by WC–Co interaction and intensification of the surface diffusion.

### 2.4. Implementation of developed DS alloys

Developed DS alloys were used as a binder for fabrication of drills with diamond segments destined for drilling reinforced concrete. Parameters of the fabricated diamond drill are presented in Table 3.

**Table 3:** Parameters of diamond drill.

	Segment geometry, mm	Segments per drill	Segment production method
Diamond drill ∅ = 100 mm	24 × 3.5 × 7	9	Hot pressing in inert atmosphere

Drilling tests were carried out on reinforced concrete with different contents of ferrous armature (*A*) that is widely used in the building industry. Variation in *A* (from 0 to 13 vol %) allowed us to change the cutting conditions from relatively easy to very hard. Cutting speed (*V*_cut_) and specific service life (*R*_s_) were calculated using formulas (1) and (2):

(1) $$V_{\text{cut}}=\frac{h}{\tau }$$

where *V*_cut_ is the cutting speed (cm/s), *h* height of concrete slab (cm), τ drilling time for path *h* (in s):

(2) $$R_{\text{s}}=\frac{L}{h_{\text{segm}}}$$

where *R*_s_ is the specific service life, *L* drilling path (m), *h*_segm_ segment wear during drilling path *L* (in mm)

**Figure 7:**
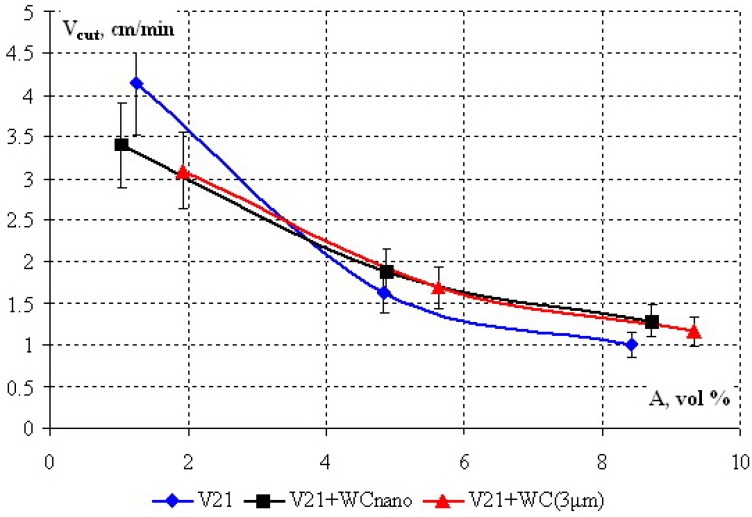
Cutting speed *V*_cut_ *vs*. armature content *A* (vol %) for diamond segments with a V21 binder dispersion-strengthened with nano- and micro-sized WC particles.

Results in Figure 7 indicate that the values of cutting speed for all investigated segments lie within the confidence interval of the experiment. This implies that insertion of reinforcing additives in the binder does not reduce the average protrusion of diamond grains above the binder. As is known, protrusion depends on the relationship between the wear rates of diamond grain and binder. For DS binder wear rate is to decrease so close cutting speed in comparison of pure V21-alloy to indicate that wear velocity of diamonds is also decrease. It means that an increase in the service life of cutting grains is possible only upon improvement of binder adhesion to diamond due to interaction between WC nanoparticles and diamond.

**Figure 8:**
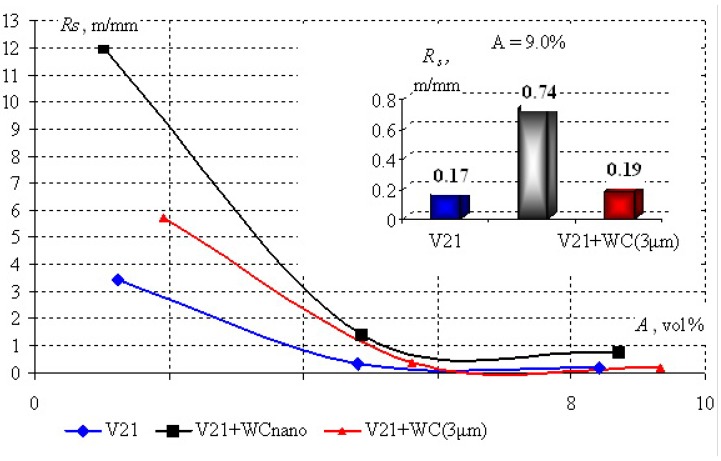
Specific service life *R*_s_ *vs*. armature content *A* (vol %) for diamond segments with a V21 binder dispersion-strengthened with nano- and micro-sized WC particles.

Figure 8 shows the specific service life *R*_s_ of the investigated diamond segments *vs*. armature content *A*. The inset to Figure 8 shows the *R*_s_ values at *A* = 9 vol %. It is seen in the Figure, the specific working life of diamond tool strongly depends on armature content. For low *A*, the drill with a V21–WC (3 μm) binder has a shorter tool life than that with a pure V21 binder. The use of a V21–WC^nano^ binder gave a 3-fold gain in the tool life.

It is important to note that positive influence of nanoparticles on *R*_s_ was observed over the entire range of *A*.

## 3. Experimental Section

The powders used in our experiments are characterized in Table 4, Table 5.

**Table 4:** Properties of starting powders.

	Particle size, μm	Composition, wt %							
		Co	Fe	Ni	Cu	W	Sn	Cr	P
Co	0.5–3	99.25	–	–	0.75	–	–	–	–
V21	2–6	15	74	–	9	–	1	–	1
B13	2–20	0.5	12	34	42	0.5	6.5	4	–

**Table 5:** Properties of added nanopowders.

	Particle size *d*, nm	Specific surface *S*_sp_, m^2^/g	Apparent density ρ_ap_, g/cm^3^	Impurities, wt %	Production method
Al_2_O_3_	10–40	13–25	0.2	0.014–0.2	Plasmochemical synthesis
ZrO_2_	10–40	10–14	0.5	0.1–0.05	Plasmochemical synthesis
WC	20–100	6–9	2.4	up to 5 %	Plasmochemical synthesis
Si_3_N_4_	10–100, fibers	10–20	0.5	up to 3 %	SHS

Starting mixtures were prepared in a centrifugal planetary mill (CPM) under controllable balls/mixture ratio and varied treatment duration. The distribution of nanoparticles over the charge bulk was investigated by Auger spectroscopy (PHI-680 Auger nanoprobe, Physical Electronics). Charges with different nanopowder contents were sintered at *T*_s_ = 800–1,000 °C. The samples for mechanical and tribological testing were obtained by hot pressing at *T* = 850–900 °C and *P* = 350 kg/cm^2^ in an inert atmosphere. Density and mechanical properties were determined for three samples, and the results of measurements were processed statistically. Microstructure was investigated by TEM (CM 200 installation, Philips). Tribological tests were performed in an automated friction machine (CSM Instruments) by “immobile small ball–rotating disc” scheme under the following conditions: The rider was an Al_2_O_3_ ball 3 mm in diameter, normal load 2 N, linear speed of rotation 10 cm/s, in air, track diameter 6.1 mm, and race *L* = 122–500 m. The wear groove (track) was characterized using a Mahr S8P profilometer. The value of wear *W* was calculated by using the formula:

(3) $$W=\frac{2\pi RS}{LF}$$

where *R* is the track radius (mm), *S* average cross section of the wear groove (track) (mm^2^), *L* the race (m), and *F* normal load (N).

## 4. Conclusions

Metal-matrix composite materials reinforced by nanoparticles were sintered using an intermixing procedure that ensured uniform distribution of nanoparticles over a starting charge. The sintering kinetics was found to depend on whether or not the interaction between added nanoparticles and matrix powder takes place (using as examples inactive ZrO_2_ and reactive WC nanoparticles). An increase in the amount of added nanoparticles leads to their aggregation and accumulation of conglomerates in the porous interparticle space of the binder, which exerts a decelerating effect on the compaction process. In hot-pressed samples, the reinforcing phase was found both in the grain body and its boundary. Dispersion-strengthened alloys showed an increase in the hardness (by 5–16 HRB), bending strength (by 54%), wear resistance (by a factor of 2–10) and a decrease in the friction coefficient (up to 4-fold). The use of designed alloys as a binder of cutting diamond tools gave a 4-fold increment in the service life of tools, without reduction in their cutting speed.
